# 3D kinematic of the thoracolumbar spine in Mangalarga Marchador horses performing the marcha batida gait and being led by hand—A preliminary report

**DOI:** 10.1371/journal.pone.0253697

**Published:** 2021-07-06

**Authors:** Samuel P. Simonato, Gustavo R. D. Bernardina, Leandro C. R. Ferreira, Amanda P. Silvatti, Kate M. C. Barcelos, Brunna P. A. da Fonseca

**Affiliations:** 1 Department of Veterinary, Universidade Federal de Viçosa, Viçosa, MG, Brazil; 2 School of Physical Education, Physiotherapy and Occupational Therapy, Universidade Federal de Minas Gerais, Belo Horizonte, MG, Brazil; 3 Department of Physical Education, Universidade Federal de Viçosa, Viçosa, MG, Brazil; 4 Department of Animal Science in the Veterinary and Animal Science School, Universidade Federal de Goiás, Goiânia, GO, Brazil; Massey University, NEW ZEALAND

## Abstract

This study aimed to provide a preliminary description of the sagittal and transverse plane kinematics of the thoracolumbar spine of Mangalarga Marchador (MM) horses performing the marcha batida gait, led in-hand. We evaluated the pattern of angular movement and the mean amplitude of six specific angles. An optoelectronic system was used for 3D kinematic analysis (19 cameras, 250 Hz). They were positioned around the horses and an acquisition volume of 16 × 4.8 × 3 meters was used. Eight retroreflective markers were fixed on the spine of the animals over thoracic vertebrae 8 (T8), 12 (T12), 15 (T15) and 18 (T18); over the lumbar vertebrae 3 (L3) and 5 (L5); over the 1st sacral vertebra (S1); and over the 1st coccygeal vertebra (CD1). Five trials, led from a halter, with three complete gait cycles were evaluated for each marcha batida horse. The 3D coordinates of the markers were filtered with a second-order, low-pass, Butterworth filter (10 Hz). Six angles: T8-T12-T15, T12-T15-T18, T12-T18-L5, T15-T18-L3, T18-L3-L5, and L3-S1-CD1 were obtained and projected in the sagittal (Flexion and Extension) and transverse (Lateral bending) planes. We calculated, for each angle to represent the spine movements, the mean and standard deviation of the range of motion (ROM, difference between the maximum and minimum values in a stride cycle). In order to describe the movement over an average stride cycle we calculated the mean curve of angle variation. The T8-T12-T15 angle presented the largest ROM in the transverse plane, while in the sagittal plane the T8-T12-T15, T12-T15-T18 and T12-T18-L5 angles presented the largest ROMs. The L3-S1-CD1 angle (lumbosacral region) presented the lowest ROM in both planes. A reduced flexion close to a neutral spine was found, predominantly during the diagonal support and in the cranial thoracic region. At the same time, the thoracolumbar region remains in an extension which is highlighted in the lumbosacral region. During the change of the support phase, the cranial thoracic region moved from a flexion to a slight extent, and the thoracolumbar region was flexed which is emphasized in the lumbosacral region. The lateral bending of the spine followed the direction of the diagonal supports. The small amplitude in the latero-lateral and dorsoventral movements of the thoracolumbar spine of MM horses during the marcha batida gait could contribute to the smooth and natural sensations experienced when riding in this gait. The lower mobility of these angles should be considered during the clinical examination of marcha batida-gaited horses.

## Introduction

Pleasure horses can show different types of gait and can be categorized into “gaited horses’’. Their movements are characterized by a four-beat rhythm and lack of a suspension period [1,2]. Marcha is one type of gaited movement, and these horses are being used for pleasure, gait competitions, halter, or conformation competition, and is an exceptionally smooth gait for the rider. An “ideal pattern” is represented with no suspension phase and is interspersed with moments of triple support, diagonal and lateral support. Marcha is divided in two sub-types in the Mangalarga Marchador (MM) breed, called “marcha batida”, that is, when the horse spends more time in the diagonal support, and “marcha picada”, that is, when the horse spends more time in the lateral and triple supports; these horses are also capable of performing the walk and canter gaits [3]. The marcha picada phenotype is apparently controlled by only one gene (DMRT3), while the marcha batida gait may be controlled by a larger number of genes in the Brazilian Mangalarga Marchador Breed [4]. Probably because of that, eight different types of marcha gait movement variations have been shown [1] in the MM breed, admitting in some types, occurrence of short moments of suspension.

These differences in gait types are divided qualitatively and visually by technicians or official judges of the breed. Therefore, they subdivided it for competitions in just two large groups, marcha batida or marcha picada, as previously described. These horses are valuable because of their movement and smoothness and begin competing in gait and halter exhibitions at 12 months of age led in-hand, and by 36 months under the saddle in more strenuous exercise [3].

The spine structure is important to support the horse’s body and give mobility to their capacity to locomote. For the horse’s locomotion, the spine is important, and when there are injuries and dysfunctions in their structures a reduction in a horse’s athletic performance could be observed. More than this, the horse can play different interactions with humans, as hippotherapy [5–7], and in this context, a correct and normal spine movement of the horse can affect, directly, the practitioner, and an increase or a decrease in the spinal range of movement could be chosen in order to treat specific syndromes in humans in the future. The occurrence of back pain in these horses can affect their welfare and their capacity to help in the treatment of some pathologies in humans’ beings.

Horses suffering from back pain are often subjected to a long period of athletic inactivity and medical treatment, often with a doubtful prognosis [8]. Thus, to understand the causes of back pain, the circumstances that lead to pain and the physical mechanism underlying these injuries are necessary to gain a comprehensive knowledge of both the functional and biomechanical anatomy of the vertebral column of horses. However, data on movements (kinematics) and forces (kinetics) that act on the horse’s back are difficult to obtain because the mobility of the spine is limited and the thoracolumbar region is composed of numerous structures that are covered with a comparatively large muscle mass [8,9].

Many in-vitro cadaveric studies have examined the mobility of the equine spine [10–13], and numerous in-vivo studies have investigated the mobility of the back in the trot [14–17]. However, no study has analyzed this movement in the marcha batida gait, which is the natural gait of many horses such as the MM, and today it is the gait most used in Brazilian gaited competitions. MM, the largest and most common horse in Brazil, and according to the Brazilian Mangalarga Marchador Horse Breeders Association, (ABCCMM) there are 600,000 registered horses and 67 regional breeder organizations in Brazil [4], and now also with some new horses in other countries. The popularity of gaited horse breeds is increasing, and accurate descriptions of limb kinematics are needed both to define the performance characteristics of the different gaits and to assist with identifying pathologies [2]. It appears logical to conclude that the movement of the horse’s spine is influenced by its type of gait [16], emphasizing the importance of documenting the normal movements of the vertebral column of the marcha batida gait in the MM horse.

The aim of this study was to provide a first description of the sagittal and transverse plane kinematics of the thoracolumbar spine in MM horses during the marcha batida gait, describing the pattern of angular movement and the mean amplitudes for six specific angles.

## Materials and methods

This work was approved by the Ethics Committee on Animal Use of the Federal University of Viçosa (Process n^o^. 25/2015) in accordance with the Veterinary Professional Ethics Code, Ethical Principles for Animal Research established by the Brazilian College for Animal Experimentation and current Brazilian legislation. Five MM horses that perform the marcha batida gait were included in the study: four females and one gelding, average body mass of 323 kg (310–346 kg), aged between 4 and 12 years (mean age 7.8 years), and performing at a low level of athletic activity (horses used just for leisure, pleasure horses). These animals were subjected to clinical examinations of both the locomotor system and the thoracolumbar area, which included inspection, palpation, mobility tests and analysis of the animal in movement. None of the animals presented with lameness or back pain.

**Fig 1:**
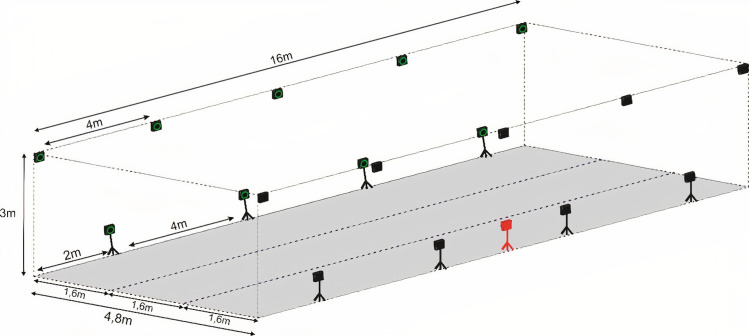
Schematic drawing of the cameras position (acquisition volume of 16 x 4.8 x 3 m^3^). The dotted lines represent the path where the animals were led.

Kinematic data were acquired using an Optitrack® motion capture system composed of 19 optoelectronic cameras (Prime17w), with an acquisition frequency of 250 Hz and connected to the software, Motive MTV-BDY. Cameras were positioned along a flat concrete floor that allows an acquisition volume of 16.0 × 4.8 × 3.0 meters (L x W x H) (Fig 1). Ten cameras were positioned approximately three meters above the ground, and nine were situated one meter above the ground.

**Fig 2:**
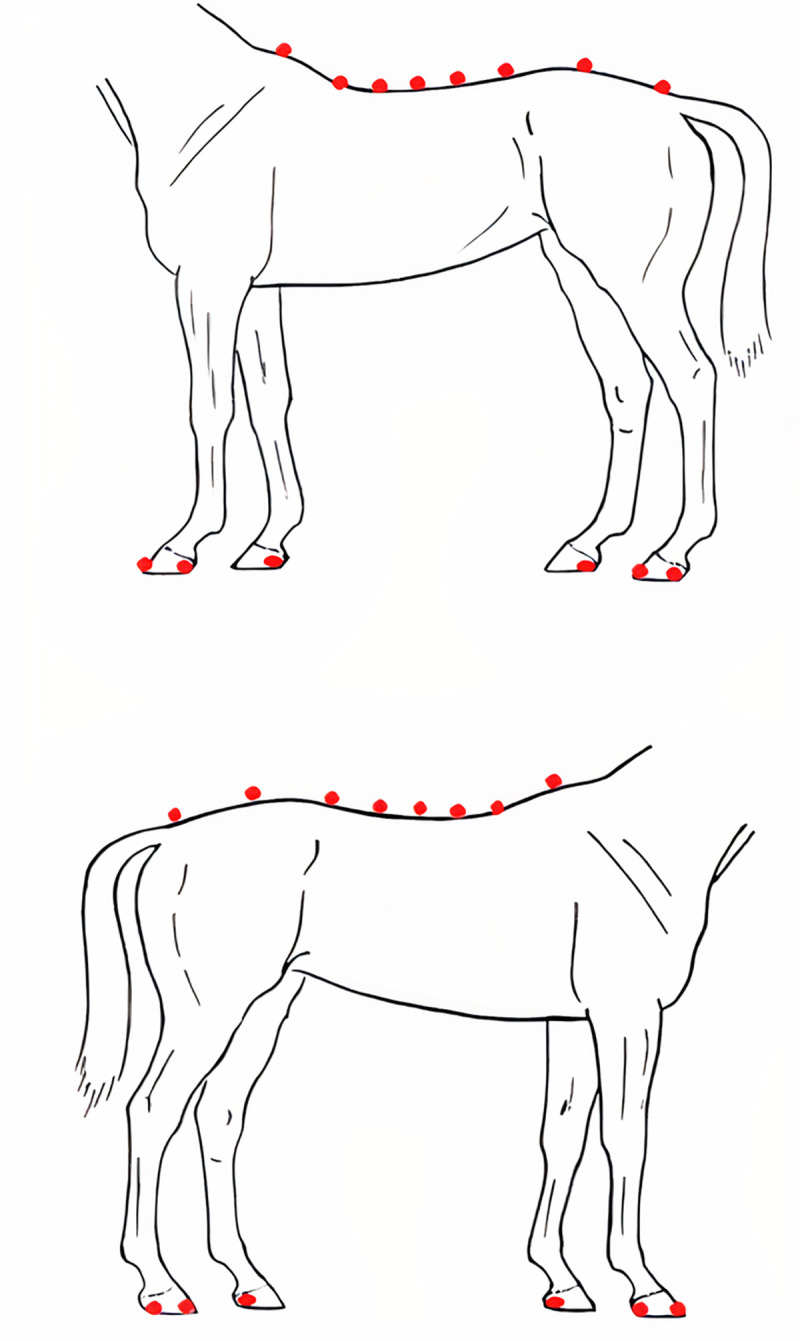
Marker’s position the fixed on the animal’s skin or hoof.

The markers used were of 2.6 cm diameter spheres made of propylene and covered by a retroreflective adhesive. They were affixed to the animal’s skin with double-sided hypoallergenic tape, in the spinous processes (SP) of the 8th (T8), 12th (T12), 15th (T15), and 18th (T18) thoracic vertebrae, the 3rd (L3) and 5th (L5) lumbar vertebrae, the 1st (S1) sacral vertebra and the 1st (CD1) coccygeal vertebra. These anatomical references were identified by palpation of the SP along the axial midline (Fig 2). Additionally, three of the same markers were fixed on the hooves of each animal: the dorsolateral end of the toe and in each heel (medial and lateral) to determine the periods of support and suspension of each limb (Fig 2).

Before the process of data acquisition, the horses underwent an adaptation period during which they were led through the data capture volume to become familiar with the procedures.

For the analysis of the data collected, a global system of 3D right-handed coordinates was created inside the volume of acquisition, in which the X-axis represented horizontal craniocaudal movement (positive cranial), the Y-axis represented vertical dorsoventral movement (positive up), and the Z-axis represented horizontal latero-lateral movement (positive left) (Fig 1).

Animals were led in-hand in the marcha batida gait with their heads free, as is common in any lameness exam, and at a self-selected speed by each animal. Each animal performed five trials with three consecutive complete strides. This allowed us to analyze 15 strides cycles for each animal.

After data collection, the 3D coordinates of the markers were processed using MATLAB software. Initially, the coordinates were filtered with a second-order low-pass Butterworth filter with 10 Hz cutoff frequency, in accordance with previous studies [16]. For the description of movements, angles between three markers that defined each region of the study were calculated. The dorsoventral movement was described by projection of the angles in the sagittal plane formed by the X and Y axes. A reduced angle represents extension; and an increased angle represents a flexion of the spine [16]. The lateral banding movement was described by the projection of the angle into the transverse plane, formed by the X and Z axes. A reduction of this angle represented the lateral movement of the spine. Where the spine bending to the left is the same as to say with the direction of the front and the back end of the horse bending to the left side, and the middle of the spine to the right side. Whereas the opposite movement is an increase of this angle that represents the spine bending to the right. In order to determine the speed of each horse, the mean speed values were calculated for each marker along the X-axis, and the results were expressed in ms^-1^.

The angles studied in this study were formed by the markers T8-T12-T15 (cranial thoracic) and T12-T15-T18 (caudal thoracic) for movements in the thoracic region; T12-T18-L5 (thoracolumbar 1) and T15-T18-L3 (thoracolumbar 2) for movements in thoracolumbar transition; T18-L3-L5 (lumbar) for movements in the lumbar region, and L3-S1-CD1 (lumbosacral) for movements in the lumbosacral region. The result of the angle calculation was time-normalized to the stride cycle by interpolation to 101 data points (0 to 100%), where 0% represents the foot contact of the right front hoof and 100% the beginning of the subsequent foot contact of the same hoof. The right and left diagonals were defined by the supports of the left thoracic and right thoracic limbs, respectively.

We calculated for each angle to represent the spinal movements, the mean and standard deviation of the range of motion (ROM, difference between the maximum and minimum values in a stride cycle).

In order to describe the movement over an average stride cycle (from the 15 strides cycles of each animal) we calculated, for each animal, the mean curve of the angle variation. This defined the angular motion pattern (AMP), which was related to both periods of support and suspension of each limb during each cycle.

## Results

**Fig 3:**
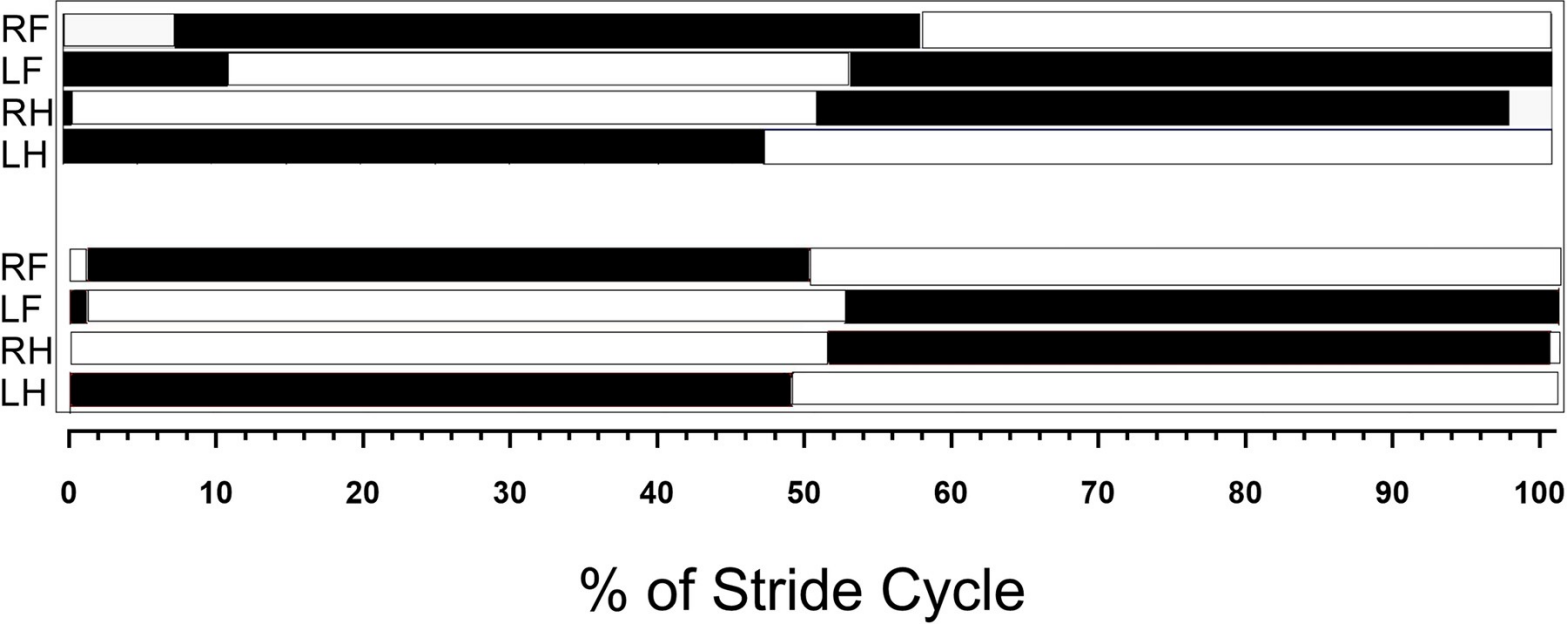
Two-footfall sequence represented in bar diagrams of 0 to 100% marcha batida gait stride cycle (black bars represent moments of support and white bars represent moments of suspension).

When evaluating the horse’s gait patterns, there were two variations observed in the gait diagrams (Fig 3). In both diagrams, the movement patterns of the spine were similar.

The average speed of these horses was 3.65 (±0.27) ms^-1^ (maximum 3.85 ms^-1^and minimum 3.34 ms^-1^). All animals tolerated the procedures well and showed no signs of discomfort with the techniques used during data collection.

### Range of Motion (ROM)

In the transverse plane (Table 1), the ROM values were bigger than in the sagittal plane (Table 2). The cranial thoracic angle presented the largest ROM in the transverse plane with a mean value of 7.3° (±2.5°).

**Table 1:** Mean (± standard deviation) range of motion (ROM) in degrees (⁰) of individual horses and all horses (General) at different angles analyzed in the transverse plane (plane X-Z).

	T8-T12-T15	T12-T15-T18	T12-T18-L5	T15-T18-L3	T18-L3-L5	L3-S1-CD1
**Horse 1**	4.4 (±1.2)	4.0 (±0.9)	3.2 (±0.5)	3.0 (±0.7)	3.0 (±0.7)	2.7 (±0.5)
**Horse 2**	9.1 (±0.9)	4.6 (±0.8)	2.8 (±0.4)	1.7 (±0.4)	4.5 (±0.3)	3.6 (±0.4)
**Horse 3**	7.4 (±1.5)	6.0 (±1.1)	5.4 (±0.9)	3.9 (1.6)	4.2 (0.6)	3.4 (0.6)
**Horse 4**	8.8 (±1.4)	4.0 (±0.8)	4.6 (±1.7)	3.9 (±0.9)	3.7 (±0.6)	3.6 (±0.5)
**Horse 5**	5.5 (±0.8)	2.2 (±0.4)	3.3 (±0.6)	2.1 (±0.3)	4.1 (±0.8)	3.1 (±0.5)
**General**	7.2 (±2.4)	4.2 (±1.4)	3.9 (±1.6)	3.9 (±1.2)	3.9 (±1.1)	3.3 (±0.8)

T8-T12-T15 = markers affixed in the 8th, 12th and 15th thoracic vertebrae; T12-T15-T18 = markers affixed in the 12th, 15th and 18th thoracic vertebrae; T12-T18-L5 = markers affixed in the 12th and 15th thoracic vertebrae and 5th lumbar vertebra; T15-T18-L3 = markers affixed in the 15th and 18th thoracic vertebrae and 3rd lumbar vertebra; T18-L3-L5 = markers affixed in the 18th thoracic vertebra and 3rd and 5th lumbar vertebrae; L3-S1-CD1 = markers affixed in the 3rd lumbar vertebra, 1st sacral vertebra and 1st coccygeal vertebra.

**Table 2:** Mean (± standard deviation) range of motion (ROM) in degrees (⁰) of individual horses and all horses (General) at different angles analyzed in the sagittal plane (plane X-Y).

	T8-T12-T15	T12-T15-T18	T12-T18-L5	T15-T18-L3	T18-L3-L5	L3-S1-CD1
**Horse 1**	2.4 (±0.5)	3.1 (±0.4)	3.3 (±0.5)	2.6 (±0.5)	2.6 (±0.4)	2.2 (±0.9)
**Horse 2**	5.0 (±0.6)	4.0 (±0.5)	3.4 (±0.4)	1.8 (±0.2)	2.2 (±0.2)	2.1 (±0.7)
**Horse 3**	3.6 (±1.0)	4.5 (±1.1)	2.6 (±0.4)	2.8 (±1.0)	2.3 (±0.5)	2.7 (±0.4)
**Horse 4**	3.0 (±0.6)	4.1 (±0.7)	3.3 (±0.5)	1.7 (±0.3)	2.0 (±0.3)	1.7 (±0.5)
**Horse 5**	1.8 (±0.3)	2.7 (±0.2)	3.5 (±0.3)	1.7 (±0.2)	2.6 (±0.4)	0.9 (±0.3)
**General**	3.2 (±1.2)	3.7 (±1.2)	3.2 (±0.7)	2.2 (±0.8)	2.3 (±0.5)	1.9 (±0.8)

T8-T12-T15 = markers affixed in the 8th, 12th and 15th thoracic vertebrae; T12-T15-T18 = markers affixed in the 12th, 15th and 18th thoracic vertebrae; T12-T18-L5 = markers affixed in the 12th and 15th thoracic vertebrae and 5th lumbar vertebra; T15-T18-L3 = markers affixed in the 15th and 18th thoracic vertebrae and 3rd lumbar vertebra; T18-L3-L5 = markers affixed in the 18th thoracic vertebrae and 3rd and 5th lumbar vertebrae; L3-S1-Cd1 = markers affixed in the 3rd lumbar vertebra, 1st sacral vertebra and 1st coccygeal vertebra.

In the sagittal plane, the caudal thoracic, thoracolumbar 1 and cranial thoracic angles presented the largest ROM in the sagittal plane, respectively, caudal thoracic 3.7° (±1.2°), thoracolumbar 3.2° (±0.7°) and cranial thoracic 3.2° (±1.2°). The lumbosacral angle presented the smaller ROM in the sagittal plane with 1.9° (±0.8°).

### Angular Motion Pattern (AMP)

#### Flexion and extension

**Fig 4:**
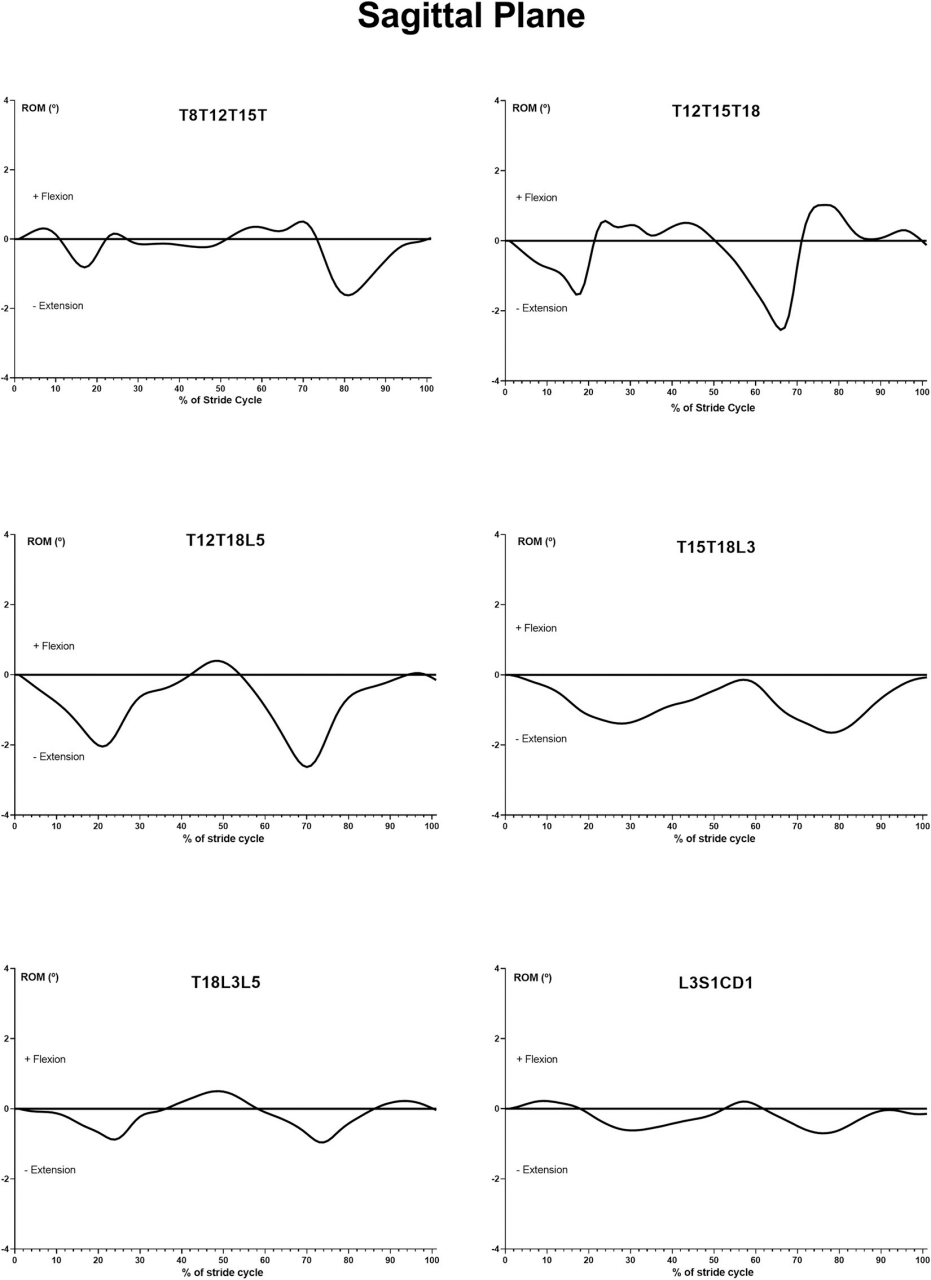
Typical example of mean curve of angular motion pattern (AMP) of the T8-T12-T15, T12-T15-T18, T12-T18-L5, T15-T18-L3, T18-L3-L5 and L3-S1-Cd1 angles in the sagittal plane, in relation to the average of both stride cycles.

In the sagittal plane (Fig 4), maximum extension of the cranial thoracic angle occurred in the 20% and 80% of stride cycle, during the first and second diagonal supports. The maximum flexion occurs in 25% of the stride cycle and keeps flexing until 75% of the stride cycle, during the period of suspension or during tripedal support, and in the early beginning of second diagonal support.

The caudal thoracic angle had a close pattern as the previous angle with the largest point of extension occurring in the 20% and 70% of the stride cycle, and the largest point of flexion in the 25% to 50% and 75% to 100% of the stride cycle, in the medium and final third of the diagonal supports (Fig 4). The change of support occurs in the beginning up until 10% of the stride cycle, and between 45% at 60% in the same cycle. In the change of supports, the caudal thoracic angle was found at the beginning of the extension.

The movement of the thoracolumbar minimum values of these angles, which correspond to maximum extension of the spine, occurred close to the 20% and 70% of the stride cycle, during diagonal support either in the thoracolumbar 1 angle or in thoracolumbar 2 angle (Fig 4). For these angles, a little flexion occurred at the end of the second diagonal support phase, and the maximum flexion during the second changeover between supports. The lumbar angle movement followed the thoracolumbar area behavior (Fig 4).

The dorsoventral movement of the lumbosacral region presented two largest points of flexion. However, the first one occurred in the opposite direction of the thoracolumbar and lumbar regions up to 10% of the stride cycle (Fig 4).

### Lateral bending

**Fig 5:**
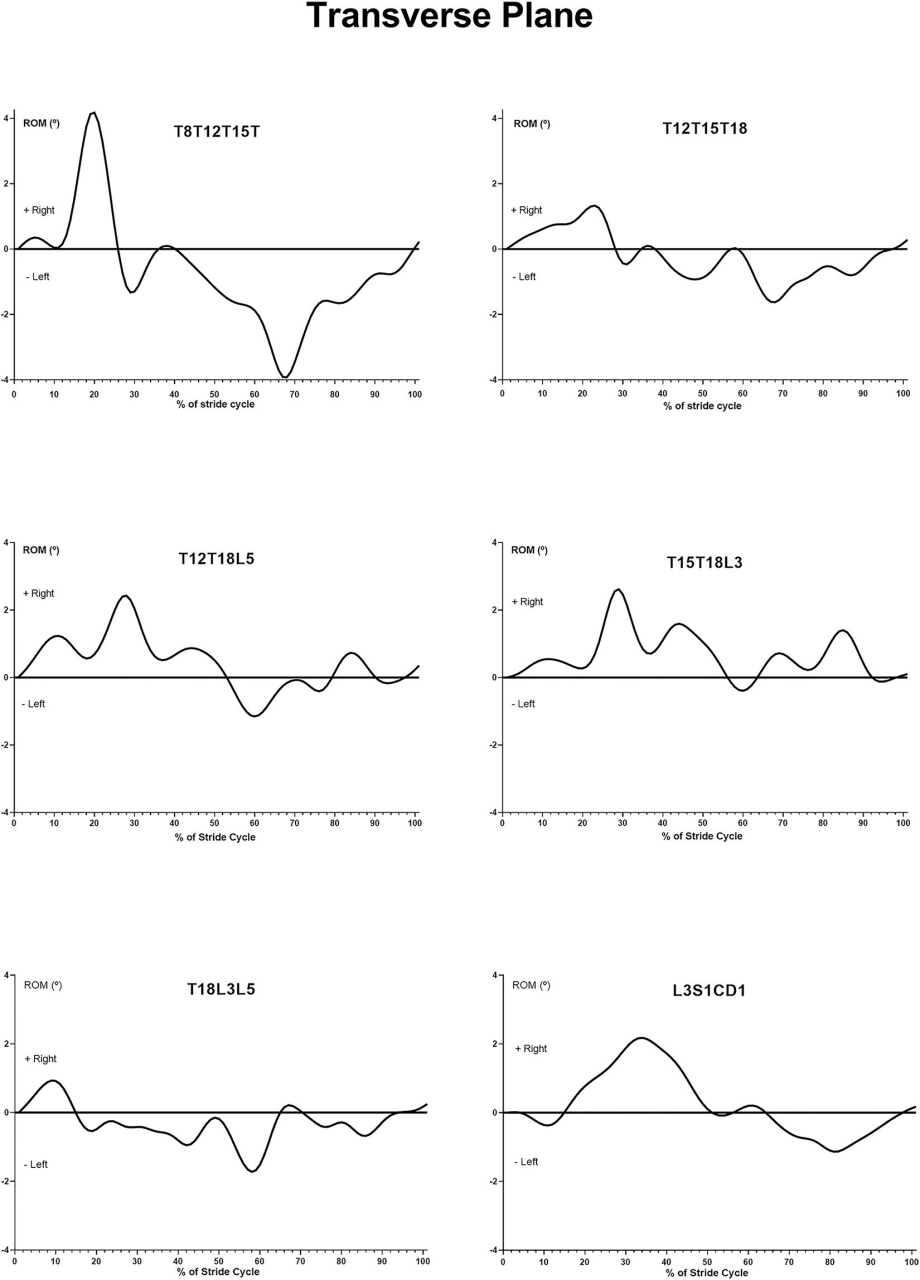
Typical example of mean curve of angular motion pattern (AMP) of the T8-T12-T15, T12-T15-T18, T12-T18-L5, T15-T18-L3, T18-L3-L5 and L3-S1-Cd1 angles, in the transverse plane, in relation to the average of both stride cycles.

The illustration of the angular motion pattern (AMP) shows that the cranial thoracic angle, i.e., the lateral bending, takes place in the 20% and 70% of the stride cycle, on the same side of the front limb support (Fig 5). The caudal thoracic angle presented the same pattern of lateral bending (Fig 5). The same occurs in the movement of the thoracolumbar area, and lumbar angle (Fig 5) in the transverse plane. Lateral movement of the lumbosacral angle in the transverse plane followed the lumbar area pattern (Fig 5).

## Discussion

### Range of Motion (ROM)

In general, our results suggest a small thoracolumbo sacral movement during the acquisition of data condition (Tables 1 and 2). In trotting horses, mainly in the lumbar region [18], greater latero-lateral movement was observed with values ranging from 3.6 to 5.4 degrees in the segments T13-T17-L1 and L1-L3-L5. Thus, when comparing with the findings in trotting horses [16,18] at similar speeds, the marcha batida gait provides reduced movement amplitudes of lateral bending and flexion-extension that could be associated with a reduced mobility in both the sagittal and transverse plane in the region of the thoracolumbar and the lumbosacral spine.

The angles in the thoracic region in the transverse plane (Table 1) are similar to those reported in [18] who evaluated six sound Dutch Warmblood horses that trotted on a treadmill and overground at their own preferred speed (mean ± s.d. 3.6 ± 0.3 ms^-1^), and the mean amplitude value overground was 4.4° (±0.9°) for T10-T13-T17 and 3.6° (±0.5°) for T13-T17-L1.

The dorsoventral movement of the cranial thoracic angle presented a lower mean value (3.2°), when compared with the T6-T12-T18 angle in trotting horses at the same [15,16] and at different [18] speeds. Based on that, our data suggest that dorsoventral movement of the cranial thoracic angle is the region of less mobility when comparing marcha batida and trot gaits.

This appears to be a region of less mobility when comparing marcha batida and trot gaits. In MM marcha batida gait horses at 3.65 ms^-1^ it was observed that the thoracic region is more stable than the same region in trotting horses [15,16]. However, one study [18] in trotting horses has reported smaller values in dorsoventral movement at the T10-T13-T17 angle when performing the trot at 3.6 ms^-1^ in a similar setup. Thus, studies with a larger number of MM horses should be undertaken considering the different types of marcha gait to elucidate this issue.

The thoracolumbar 1 dorsoventral ROM angles were observed for other researchers with a similar value of 3.5° ROM at an angle formed by the T12-T18-S1 vertebrae in horses trotting on a track in the conditions of the routine lameness examination at 3.16 ms^-1^ [15], and an amplitude of 2.2° for the T13-T17-L1 angle [18], similar to that found in the thoracolumbar 2 in this study.

The differences in the ROM between marcha batida gait and trot suggest that the marcha batida gait in both variations evaluated in this study, is related to a particular movement in the MM thoracolumbar spine maintaining a flexed posture, during diagonal supports observed for the thoracic region. This is likely related to differences in the footfall sequence of limb support between the marcha batida gait found in this work (Fig 3) and the trot [16].

It was observed that the ROM of the lumbar angle (2.3°) was lower than that found by other researchers at angles T17-L1-L3 (3.2°) and L1-L3-L5 (4.2°) in trotting horses [18]. This difference may be associated with the marcha batida gait by keeping the horse in continuous contact with the ground for a longer time. By doing this, it transmits reduced vertical impact forces to the spine [8].

The lumbosacral region of the MM horses, represented by the angle L3-S1-CD1, presented 1.9° of the range of movement related to the flexion-extension (Table 2). Three studies in trotting horses, Dutch Warmblood [16,18] and Selle Français and Trotteur Français [19] showed mean amplitude values of 3.8°, 3.7°, and 3.9°, respectively [16,18,19]. These values were higher than the values found in our paper. It is assumed that the dissociation in support exchanges, with or without a short suspension time period in marcha batida gait, was necessary for the MM to express its marcha batida gait, that could be influencing the movement of the lumbosacral joint as well as the absence of long suspension time, which is the opposite of what happens in trotting animals.

### Angular Motion Pattern (AMP)

#### Flexion and extension

In the sagittal plane, the angles of the thoracic region present two moments of maximum extension during the stride cycle, intercalated by plateaus of flexion. In the caudal thoracic angle, moments of maximum extension were observed earlier in the stride cycle compared to the cranial thoracic angle. The moment when the cranial thoracic angle reaches the maximum extension, it differs from those reported by other researchers [15] in trotting horses the dorsoventral movement of a similar angle (T6-T13-L1). In trotting horses, the maximum extension occurs during diagonal support, but early 10% in the stride cycle. Other researchers [17] reported horses in trot where the maximum extension occurred at a similar time to that of the cranial thoracic angle (T6-T13-L1) in the present study (20% of stride cycle).

A particularity observed in MM horses was the presence of plateaus of flexion of the spine, which was not reported in trotting horses studied by others [15,19]. This difference suggests that postural alterations occur in MM horses to maintain the stability of the spine, especially prior to the exchange of supports, which does not always have a suspension time. When the suspension time occurs, this is minimal, which reflects differences in the movement of the spine between the marcha batida and the reports of trotting animals.

The maximum extension of the thoracolumbar region occurred at the same moments as trotting horses, in the 20% and 70% of stride cycle, during the middle third of diagonal support, as studied by other researchers [15,19]. It is worthy to note that at these angles, although less evident than those in the thoracic region, there is a tendency to maintain the flexion posture in the spine, which reinforces the hypothesis of the importance of this movement for MM horses. When the muscles exert greater force, pressing the vertebrae more firmly together and stiffening the back, the spine acts as a transmitter of the impulse generated by the pelvic limbs to the rest of the horse’s body [19], which allows the horse to start or continue the movement. Some riders of gaited horses remark on how comfortable their breed’s special gait feels, compared with the trot [20]. So, we believe that this pattern of spine movement could contribute to providing horizontal displacement with reduced transmission of vertical impact to the spine.

In the sagittal plane, we observed that both flexion and extension of the lumbosacral joint occurred opposite to the thoracic cranial region. The predominance of the extension found in the MM has not been reported in trotting animals, which present a characteristic sinusoidal pattern [15,19]. The possible explanation for this behavior of the lumbosacral joint may be related to the dissociation of the hind limbs in relation to the front limbs to sustain the marcha batida gait. Thus, to develop the marcha batida gait, horses must maintain the support of the hind limb while their diagonal forelimb begins the suspension phase. Therefore, the extension of the lumbosacral joint along with the other peculiarities observed in the entire trunk may be a determining factor in the execution of this movement.

#### Lateral bending

The lateral movement follows the same direction as the diagonal supports in all segments. This means that when we observe the right diagonal (right forelimb and left hindlimb), we have lateral bending to the right, and when we observed at the left diagonal (left forelimb and right hindlimb), we have lateral bending to the left.

The AMP of the cranial and caudal thoracic angles in the transverse plane, both presented the same behavior of lateral bending, ipsilateral to the supporting forelimb, in the 20% and 70% of the stride cycle, during the middle third part of the diagonal support phase (Fig 3). This behavior is similar to that found in trotting horses in other studies [16,17]. The lateral movement of the spine in the thoracic region is influenced by the supporting phases of the forelimbs. During the first half of the stance phase, the dorsal part of the back tends to move toward the non-supported limb.

The AMP that describes the movement of the thoracolumbar regions 1 and 2, and the lumbar region presented similar movements. This movement includes lateral bending in the 20% and 70% of stride cycle, the middle third part of the diagonal support phase and ipsilateral to the supported hind limb (Fig 5). These findings are already described by other authors for trotting horses [16,17]. From the middle third part of the support phase, the hind limbs generate impulsion through production of ground reaction forces, exerting an oblique force on the spinal column with the lateral component pushing the spine, causing lateral bending towards the side of the supported hind limb [16]. This mechanism seems to be shared by the MM and trotting horses since both generate impulsion for the displacement of the body in the hind limbs.

The lumbosacral angle presents an AMP (Fig 5) in the transverse plane with two high points of lateral flexion ipsilateral to the supporting hind limb, occurring in the 10% and 60% of stride cycle, during the middle third of the support phases of the hind limbs. This behavior is related to the impulse of the hind limbs, as discussed earlier.

### Limitations of this study

Some limitations were identified during the development of this work. Since we did not use a local coordinate system, we failed to create a reference axis for latero-lateral movement, which would help to describe this movement. As an alternative, we could have acquired a static model of each animal, to obtain a reference value for each angle studied, and then perform the description of movements in both the sagittal and transverse planes.

Animals evaluated in this study had speed values higher than desired for medium velocity of competition standard of the breed (3.33 ms^-1^). This is another limitation of our study that may have impaired, but not precluded, the development of characteristics of the marcha gait in the animals studied, as well as types previously reported [1]. It is reported in trotting horses that increased speed can lead to reduced spinal ROM [19]. Another author [21] showed that the increase in speed decreased the time of triple supports until their absence, consequently increasing the time of diagonal bipedal supports, reaching higher speeds resulting in the occurrence of monopedal support and suspension in the marcha gait, which could exacerbate the differences between the trot and marcha batida gaits.

## Conclusion

In the marcha batida gait, differences were observed in the variation of spinal angles along the stride cycle, especially for angles of the thoracic region which showed a plateau in the flexion-extension movement curve indicating that these horses assume a posture of a small flexion close to neutrality of the spine, predominantly during diagonal support mainly in the cranial thoracic region. The lateral bending and flexion-extension angular movements of the thoracolumbar spine of Mangalarga Marchador horses during the marcha batida has, in general, small amplitude, and should be considered during the lameness examination of marcha batida gaited horses so that there is no clinical interpretation error. Furthermore, all these movements observed along the backs of Mangalarga Marchador horses when ridden could be a factor to contribute for the smoothness of this gait, mentioned in the standard of the breed.

## Supporting information

S1 File (RAR)
